# Aging aggravates acetaminophen-induced acute liver injury and inflammation through inordinate C/EBPα-BMP9 crosstalk

**DOI:** 10.1186/s13578-023-01014-6

**Published:** 2023-03-21

**Authors:** Rui Liu, Wentao Xu, He Zhu, Zijian Dong, Huke Dong, Shi Yin

**Affiliations:** 1 Department of Geriatrics, Affiliated Provincial Hospital of Anhui Medical University, Anhui Medical University, Hefei, 230001 People’s Republic of China; 2 Department of Geriatrics, Division of Life Sciences and Medicine, The First Affiliated Hospital of USTC, University of Science and Technology of China, Hefei, 230001 Anhui China; 3 Department of Oncology, The First Affiliated Hospital of Anhui Medical University, Hefei, 230022 China; 4 Clinical Medical College of Anhui Medical University, Hefei, 230036 China

**Keywords:** Aging, Macrophage, Bone morphogenetic protein 9, CCAAT/enhancer binding protein α, Autophagy, Inflammation

## Abstract

**Background:**

Previous studies have shown that bone morphogenetic protein 9 (BMP9) is almost exclusively produced in the liver and reaches tissues throughout the body as a secreted protein. However, the mechanism of BMP9 action and its role in aging-associated liver injury and inflammation are still unclear.

**Results:**

Aging significantly aggravates acetaminophen (APAP)-induced acute liver injury (ALI). Increased expression of CCAAT/enhancer binding protein α (C/EBPα) and BMP9 was identified in aged livers and in hepatocytes and macrophages (MФs) isolated from aged mice. Further analysis revealed that excess BMP9 was directly related to APAP-induced hepatocyte injury and death, as evidenced by activated drosophila mothers against decapentaplegic protein 1/5/9 (SMAD1/5/9) signaling, an increased dead cell/total cell ratio, decreased levels of ATG3 and ATG7, blocked autophagy, increased senescence‐associated beta‐galactosidase (SA‐β‐Gal) activity, and a higher rate of senescence‐associated secretory phenotype (SASP) acquisition. In contrast, *Bmp9* knockout (*Bmp9*^*−/−*^) partially alleviated the aforementioned manifestations of BMP9 overexpression. Moreover, BMP9 expression was found to be regulated by C/EBPα in vitro and in vivo. Notably, BMP9 also downregulated autophagy through its effect on autophagy-related genes (ATG3 and ATG7) in MΦs, which was associated with aggravated liver injury and SASP acquisition.

**Conclusions:**

In summary, the present study highlights the crucial roles played by C/EBPα-BMP9 crosstalk and provides insights into the interrelationship between hepatocytes and MΦs during acute liver injury.

**Supplementary Information:**

The online version contains supplementary material available at 10.1186/s13578-023-01014-6.

## Background

The liver plays a crucial role in the catabolism of nutrients and toxins and is considered to be a vital barrier against complex pathogenic factors entering the body from the external environment [1]. Physiologically, liver injury can be repaired by hepatocytes in young and healthy livers, as they contribute to the recovery of liver mass and function [2]. However, after excessive hepatocyte necrosis, the liver loses its regenerative capacity, leading to acute liver injury (ALI) and sometimes acute liver failure (ALF). The loss of liver regeneration capacity and injury resistance are the most prominent aging-associated alterations [3]. The aged liver undergoes extensive histological and molecular biological changes, such as altered hepatocyte morphology, reduced mitochondrial quantity and quality, and an increased proportion of multinucleated cells [4, 5]. Moreover, a typical feature of aging is a long-term chronic inflammatory response, and the aged liver produces a large amount of senescence‐associated secretory phenotype (SASP) factors, such as tumor necrosis factor-alpha (TNF-α), interleukin-1 beta (IL-1β), IL-6, and monocyte chemoattractant protein-1 (MCP-1) [6]. These alterations affect the morphology and normal physiological functions of the liver; therefore, the aged liver is sensitive to various factors that cause disease, including exogenous pathogenic factors, leading to a series of complex clinical problems.

Hepatocytes and abundant nonparenchymal cells constitute a complex intrahepatic microenvironment. The high rate of macrophage (MФ) recruitment to the liver is a hallmark feature of liver injury [7, 8], and the roles of these MФs depend on the recognition of relevant signals that drive their polarization in association with inflammation, such as cytokines, chemokines, and damage-associated molecular patterns (DAMPs) [9]. In the liver, an appropriate degree of inflammation helps to stimulate hepatocyte regeneration and self-repair, but excessive inflammation induces downstream proinflammatory cascades that promote hepatocyte injury and death [10]. Notably, MΦs can be reprogrammed to adapt to the body challenged by different immune environments, and interactions MΦs and hepatocytes undergo a plethora of interactions [11, 12]. Although innate immune responses represented by MΦs have been suggested to be essential in liver defense mechanisms, aging-associated responses of hepatic immune and nonimmune cells are not fully understood.

The bone morphogenetic protein (BMP) family is a subfamily of the transforming growth factor-beta (TGF-β) superfamily. BMP9, also known as growth differentiation factor 2 (GDF2), is a secretory protein in the BMP family and is expressed almost exclusively in the liver, where it acts in a paracrine and autocrine manner [13, 14]. In the liver, BMP9 is thought to inhibit mainly hepatocyte proliferation and to promote liver fibrosis [15]. Notably, BMP9 enhanced the proinflammatory phenotypic transformation of MΦs through an NF-κB-dependent pathway and promoted methionine- and choline-deficient diet-induced nonalcoholic steatohepatitis in mice [16]. Moreover, BMP2, a member of the BMP family, has been reported to promote colon cancer progression through the macroautophagy/autophagy pathway. However, few reports on the effect of BMP9 on autophagy in the liver are available, and the overall regulatory roles played by BMP9 in mouse liver aging, such as its effect on autophagy, hepatocyte survival after injury, and the regulation of the immune-inflammatory response, are not fully understood.

The present study revealed a C/EBPα-BMP9 axis in the liver. We investigated the cause of the increased BMP9 level in the mouse liver during aging and the potential related mechanisms in hepatocytes and MΦs. The aged liver produces excess BMP9, which downregulates ATG3 and ATG7 expression, inhibits autophagy, promotes hepatocyte injury, and drives MΦ proinflammatory activation, further aggravating liver injury. Moreover, BMP9 deletion relieved autophagy suppression, reduced proinflammatory SASP production, and attenuated acetaminophen (APAP)-induced acute liver injury (APAP-ALI) in vivo and in vitro. In addition, the expression of BMP9 was regulated by C/EBPα, which was abundantly expressed in the aged liver, possibly explaining the reason for the great increase in BMP9 expression in the aged liver.

## Results

### Aging accelerates APAP-ALI and liver inflammation

**Fig. 1:**
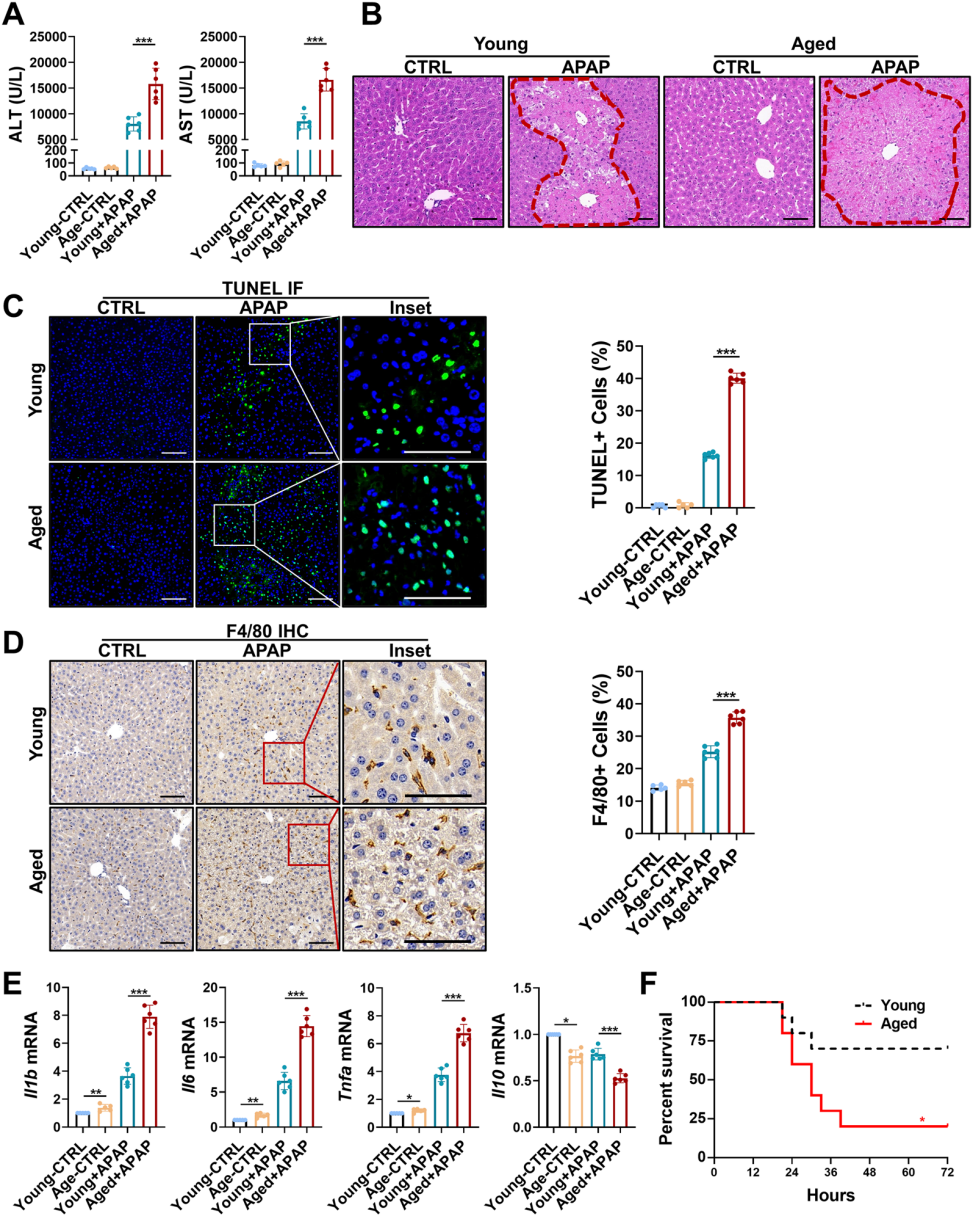
Aging accelerates APAP-ALI and inflammation. Young and aged mice were administered APAP (300 mg/kg) or PBS and analyzed 24 h later. **A** Serum ALT and AST levels of the young and aged mice treated with PBS or APAP. **B** Representative images showing H&E staining liver slices from young and aged mice treated with PBS or APAP. **C** Representative images showing TUNEL staining in the liver slices from young and aged mice treated with PBS or APAP. Quantitative data are shown in the right panel. **D** Representative images showing IHC staining for F4/80 in liver slices from young and aged mice treated with PBS or APAP. Quantitative data are shown in the right panel. **E** mRNA expression (*Il1b*, *Il6*, *Tnfa*, and *Il10*) in the livers from young and aged mice treated with PBS or APAP as measured by RT‒qPCR. The average target gene/*Gapdh* ratios of different experimental groups relative to the control group are reported. **F** Survival curves of young and aged mice treated with APAP when the dose of APAP was increased to 500 mg/kg. *p < 0.05, **p < 0.01, and ***p < 0.001

We first examined the effect of aging on APAP-ALI. The analysis revealed that aging significantly aggravates liver injury induced by APAP, as manifested by increased serum ALT and AST levels (Fig. 1A) and increases in necrotic tissue areas, as indicated by H&E staining (Fig. 1B). Moreover, the number of the terminal deoxynucleotidyl transferase (TdT)-mediated dUTP nick-end labelling (TUNEL) positive cells was significantly increased in liver tissue sections of aged mice (Fig. 1C). In addition, increased MΦ infiltration was found in the livers of aged mice after APAP-ALI had been induced (Fig. 1D). Consistent with these observations, aging promoted the expression of proinflammatory cytokines, such as SASP components (*Il1b*, *Il6*, and *Tnfa*), and decreased the expression of the anti-inflammatory cytokine *Il10* in the liver (Fig. 1E). The severity of APAP-induced liver injury was dose dependent. In a survival study, the mortality was significantly increased in the aged mice that received a higher dose of APAP (500 mg/kg) (Fig. 1F). These results indicate that aging greatly exacerbates APAP-ALI and this increase in ALI severity is accompanied by increased infiltration of MΦs and higher proinflammatory cytokine expression.

### BMP9 promotes hepatocyte injury and senescence

**Fig. 2:**
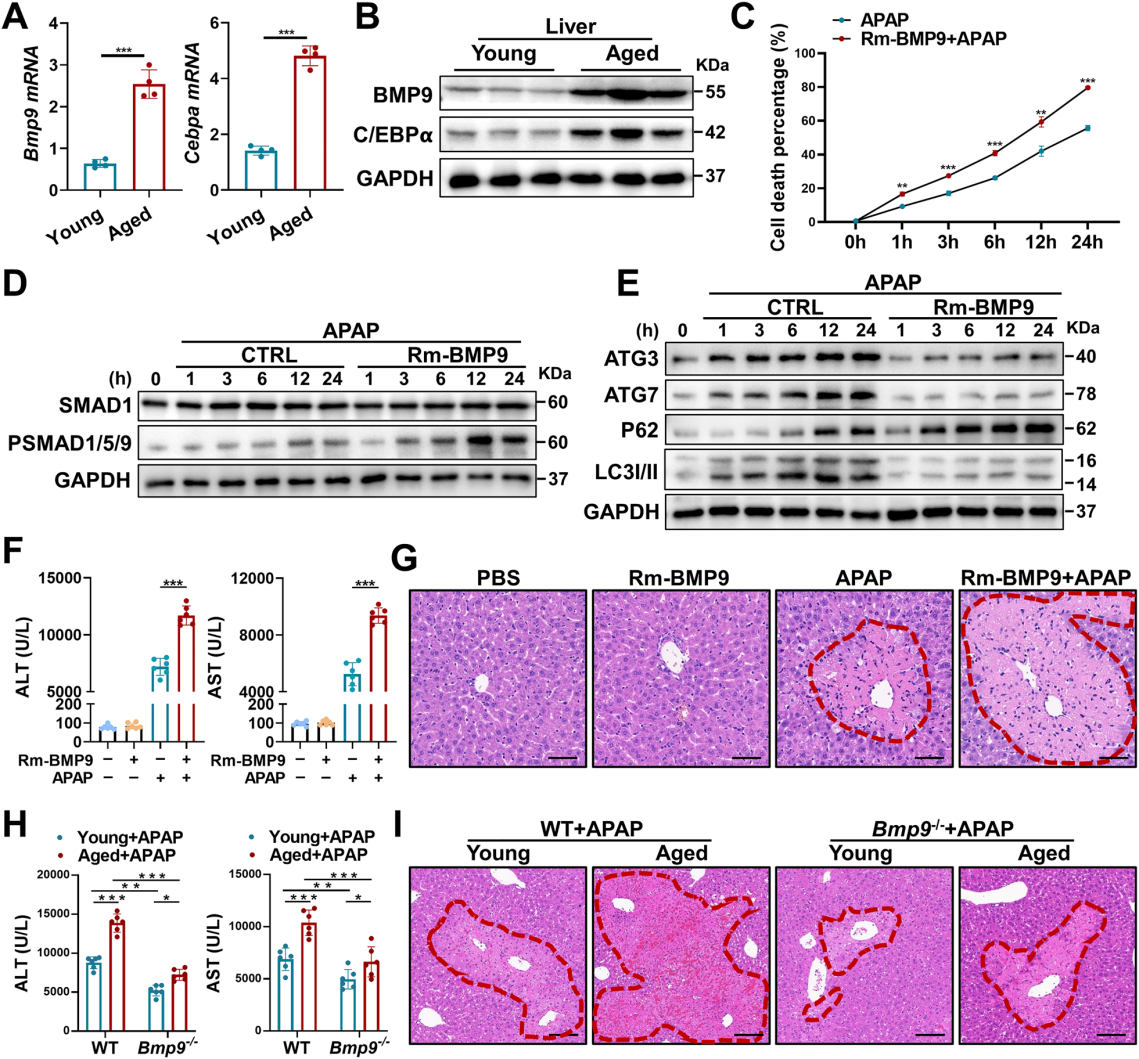
BMP9 promotes hepatocyte injury and senescence. **A**, **B** mRNA (**A**) and protein (**B**) expression in the livers from young and aged mice. (**C**–**E**) Primary hepatocytes with or without Rm-BMP9 addition were treated with APAP, and 1 h, 3 h, 6 h, 12 h and 24 h later, we measured the proportion of APAP-induced hepatocyte death to normal growing primary hepatocytes (**C**), the protein expression levels of SMAD1/5/9 and P-SMAD1/5/9 (**D**), and the protein expression levels of ATG3, ATG7, p62 and LC3 I/II (**E**). **F**, **G** Rm-BMP9 was injected into the tail vein, and the APAP-ALI model (300 mg/kg) was established 1 h later. Serum and liver tissue were collected from each group of mice 24 h after model establishment. We measured the serum ALT and AST levels (**F**), and representative images showing H&E staining (**G**) in each group are shown. **H**, **I** APAP-ALI model (300 mg/kg) was established with young and aged WT mice, as well as with young and aged *Bmp9*^*−/−*^ mice. Serum and liver tissue were collected from each group of mice 24 h later. We measured the serum ALT and AST levels (**H**), and representative images showing H&E staining (**I**) in each group are shown. The average target gene/*Gapdh* ratios of different experimental groups to the control group are reported. *p < 0.05, **p < 0.01, and ***p < 0.001

RT‒qPCR- and immunoblot-based measurements revealed that the expression of both *Bmp9* and *Cebpa* was increased in the liver tissue of aged mice (Fig. 2A, B). Since BMP9 is a secreted protein and enriched in the aged liver, we used mouse BMP9 recombinant protein (Rm-BMP9) to investigate the effect of BMP9 on APAP-treated hepatocytes. With prolonged APAP treatment in vitro, the ratio of dead-to-live hepatocytes after Rm-BMP9 treatment was significantly higher than that after APAP treatment alone, indicating that BMP9 might play a direct role in promoting hepatocyte injury induced by APAP (Fig. 2C). We further explored the mechanism by which BMP9 aggravates hepatocyte injury in vitro. Increased phosphorylation of SMAD1/5/9, representing the activation of BMP signaling, suggested that BMP9 may have activated downstream signaling cascades in hepatocytes after APAP-induced injury (Fig. 2D). Additionally, the activation of SMAD1/5/9 was increased in the livers of the aged mice, and the phosphorylation rates of SMAD1/5/9 were decreased in the livers of aged *Bmp9-*knockout (*Bmp9*^*−/−*^) mice (Additional file 1: Figure S1A).

Autophagy-related 3 (ATG3, with E2-like enzyme activity) and ATG7 (with E1-like enzyme activity) are key molecules in autophagy, mediating the transformation of microtubule-associated protein 1 light chain 3 II (MAP1LC3 II/LC3 II) and autophagosome maturation [17, 18]. Notably, Rm-BMP9 downregulated ATG3 and ATG7 expression and significantly decreased the transformation of LC3 I to LC3 II in APAP-injured hepatocytes, results that were consistent with the accumulation of SQSTM1/p62 (Fig. 2E).

These results illustrate the aggravating effect of BMP9 on aging-associated APAP-ALI in vitro, and therefore, we further investigated the effect of BMP9 on APAP-ALI in vivo by injecting Rm-BMP9 into mice. The serum ALT/AST levels (Fig. 2F) and H&E staining results (Fig. 2G) indicated significantly increased APAP-ALI severity, and the number of TUNEL- stained cells was increased (Additional file 1: Figure S1B). Then, we found that Rm-BMP9 promoted the expression of *Ilb*, *Il6* and *Tnfa* in the mouse livers (Additional file 1: Figure S1C). Consistent with the results obtained from exogenous supplementation of BMP9 in mice, *Bmp9* deletion reduced the severity of the liver injury in both young and aged mice (Fig. 2H, I). Additionally, the number of TUNEL-stained cells in the livers of young and aged mice treated with APAP was significantly decreased in the *Bmp9*^*−/−*^ groups (Additional file 1: Figure S1D). RT‒qPCR assays revealed that *Bmp9* deletion reduced the expression of proinflammatory factors (*Ilb*, *Il6* and *Tnfa*) in liver tissue (Additional file 1: Figure S1E).

These results suggest that BMP9 inhibits hepatocyte autophagy through ATG3 and ATG7, thereby regulating the severity of APAP-ALI. The high level of BMP9 protein associated with aging may be an important factor in the aggravation of APAP-ALI during aging.

### The expression of BMP9 is regulated by C/EBPα in vitro

**Fig. 3:**
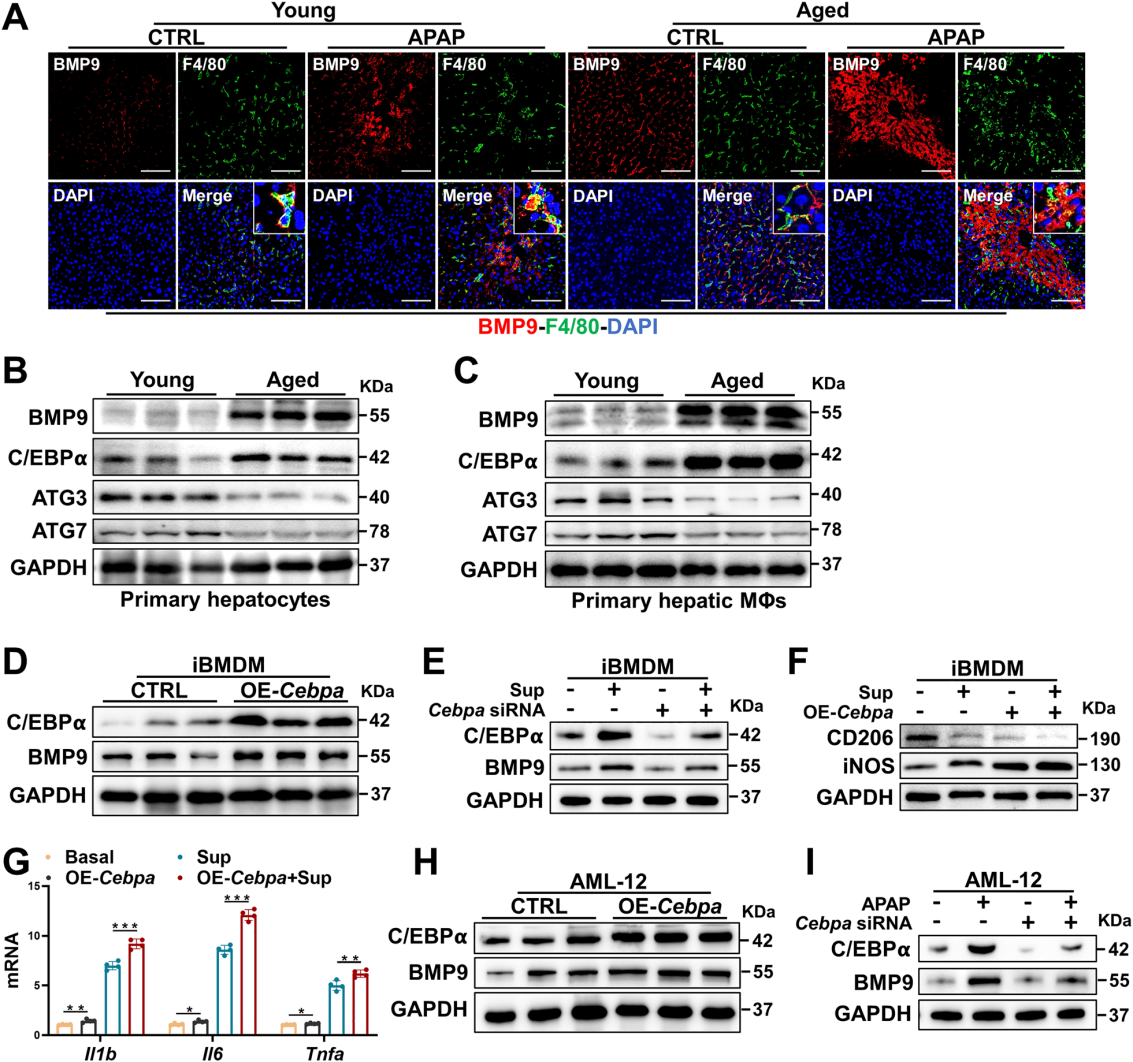
BMP9 expression is regulated by C/EBPα in vitro. **A** IF staining for BMP9 and F4/80 in liver slices from young and aged mice treated with PBS or APAP (300 mg/kg) for 24 h. **B**, **C** Primary hepatocytes and MΦs were isolated from young and aged mice by in situ perfusion of mouse livers. Protein expression levels of BMP9, C/EBPα, ATG3 and ATG7 in mouse primary hepatocytes (**B**) and hepatic MΦs (**C**). **D**–**G** To study the regulation of BMP9 by C/EBPα in MΦs, we altered *Cebpa* expression in iBMDMs. **D** Protein expression levels of C/EBPα and BMP9 after overexpressing *Cebpa*. **E** Protein expression levels of C/EBPα and BMP9 after inhibiting *Cebpa* with or without treatment with the APAP-treated AML-12 cell supernatant (Sup). **F** Protein expression levels of CD206 and iNOS after overexpressing *Cebpa* with or without Sup treatment. **G** mRNA expression of related cytokines (Il1b, Il6 and Tnfa) after overexpressing *Cebpa* with or without Sup treatment. (**H**, **I**) To study the regulation of BMP9 by C/EBPα in hepatocytes, we altered Cebpa expression in AML-12 cells. **H** Protein expression levels of C/EBPα and BMP9 after overexpressing *Cebpa*. **I** Protein expression levels of C/EBPα and BMP9 after inhibiting *Cebpa* with or without Sup treatment. The average target gene/*Gapdh* ratios in different experimental groups relative to the control group. *p < 0.05, **p < 0.01, and ***p < 0.001

Because the cell type that expresses BMP9 has not been clarified in previous studies, we sought to visualize the expression and distribution of BMP9 in aged livers. Therefore, we performed immunofluorescence (IF) staining for F4/80 and BMP9 in liver tissues of young and aged mice. In uninjured livers, BMP9 was mainly localized to F4/80-positive cells, and BMP9 expression was higher in aged mouse livers (Fig. 3A). In mice with APAP-ALI, BMP9 accumulated in injured area, BMP9 expression localized to F4/80-positive cells was increased, and BMP9 expression, in general, was identified in hepatocytes (Fig. 3A). Then, we isolated hepatocytes and hepatic MΦs from young and aged mice. We found that BMP9 expression was markedly upregulated in both the hepatocytes and hepatic MΦs from the aged mice (Fig. 3B, C). Notably, we also detected increased C/EBPα and decreased ATG3 and ATG7 levels in the isolated hepatocytes and hepatic MΦs from the aged mice (Fig. 3B, C).

C/EBPα is an important transcription factor that is a determinant in multiple physiological functions. High expression of C/EBPα and BMP9 was detected in both senescent hepatocytes and MΦs; however, whether BMP9 was regulated by C/EBPα remained unclear. Concomitant with *Cebpa* overexpression (OE), BMP9 expression was increased in the immortalized murine bone marrow-derived MΦ (iBMDM) cell line (Fig. 3D). Similarly, *Cebpa* expression in iBMDMs that was downregulated by siRNA reduced BMP9 expression (Fig. 3E). BMDMs derived from young and aged mice were then stimulated with APAP-treated AML-12 cell supernatant (Sup) to mimic DAMP signaling induced by hepatocyte death. Moreover, enhanced *Cebpa* expression promoted iNOS expression and decreased CD206 expression in the iBMDMs stimulated by Sup (Fig. 3F). Following *Cebpa* overexpression, the mRNA expression of *Il1b*, *Il6*, and *Tnfa* induced by Sup was increased in the iBMDMs (Fig. 3G). In addition, *Cebpa* overexpression increased the BMP9 level in AML-12 cells (Fig. 3H), and downregulated *Cebpa* expression correspondingly led to reduced BMP9 expression (Fig. 3I).

These results suggest that the expression of BMP9 is driven by C/EBPα signaling in MΦs and hepatocytes during aging, which might be a key intrinsic driver of aggravated APAP-ALI associated with aging.

### The C/EBPα-BMP9 axis in hepatocytes promotes APAP-ALI progression

**Fig. 4:**
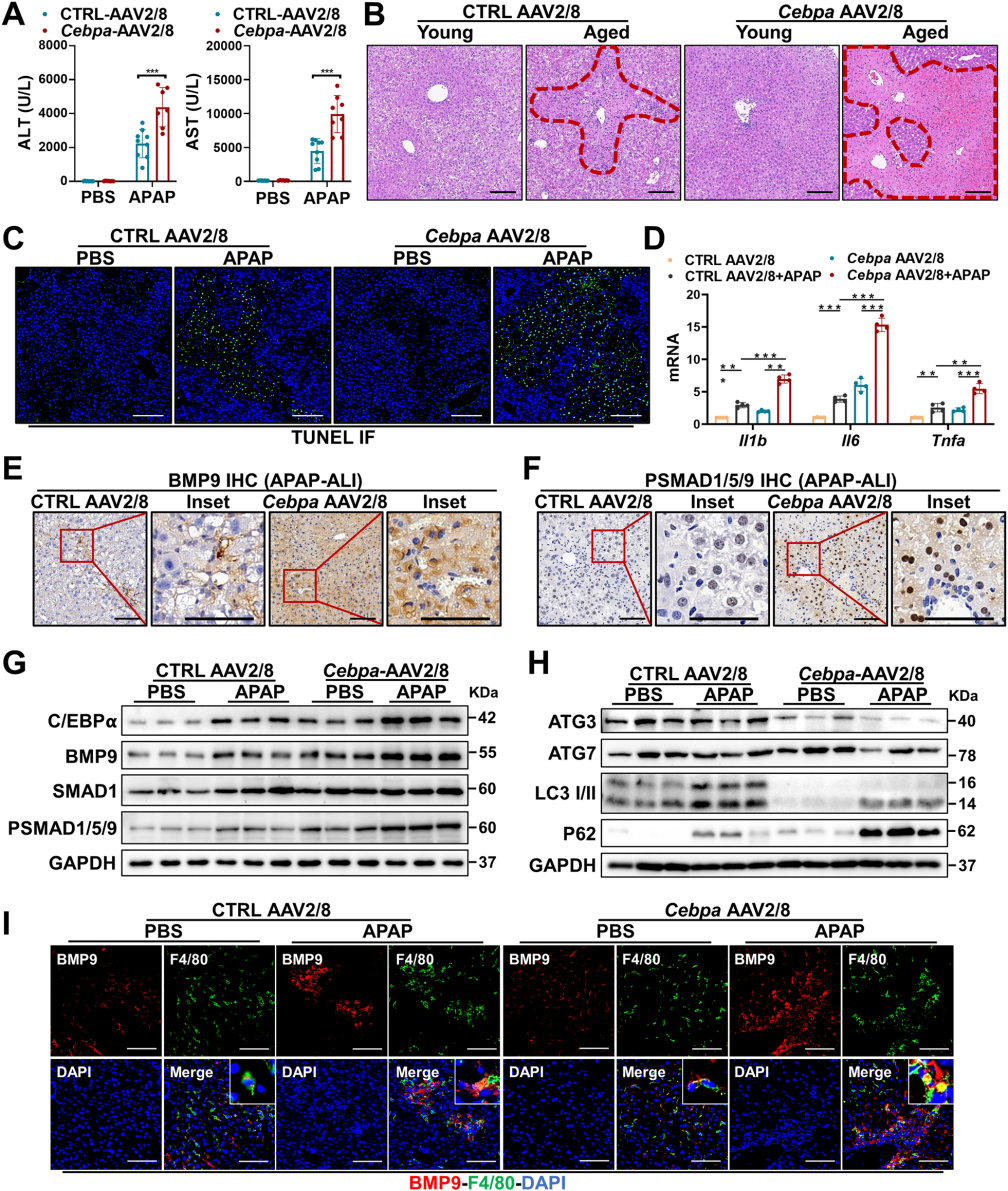
The C/EBPα-BMP9 axis in the liver promotes APAP-ALI progression. APAP-ALI (300 mg/kg) was established 2 weeks after confirmation that *Cebpa* was successfully overexpressed by *Cebpa*-overexpressing AAV2/8 injection into the tail vein of the mice, and serum and liver tissue were collected from each group of mice after 24 h. **A** Serum ALT and AST levels. **B** Representative images showing H&E staining. **C** Representative images showing TUNEL staining images. **D** mRNA expression of *Il1b*, *Il6* and *Tnfa*. **E** Representative images showing IHC staining for BMP9. **F** Representative images showing IHC staining for P-SMAD1/5/9. **G** Protein expression levels of C/EBPα, BMP9, SMAD1 and P-SMAD1/5/9. **H** Protein expression levels of ATG3, ATG7, LC3 I/II and p62. **I** Representative images showing IF staining for BMP9 and F4/80 in the liver slices of each group. The average target gene/*Gapdh* ratios different experimental groups relative to the control group. *p < 0.05, **p < 0.01, and ***p < 0.001

Given the link between C/EBPα and BMP9, the effect of C/EBPα on BMP9 expression in hepatocytes and APAP-ALI in vivo was investigated by overexpressing *Cebpa* via AAV2/8. Both the mRNA and protein expression levels of *Bmp9* were upregulated after *Cebpa* was overexpressed in the liver 2 weeks after *Cebpa* AAV2/8 injection into the tail vein (Additional file 1: Figure S2A, B). Notably, IF and IHC assays confirmed successful *Cebpa* overexpression (Additional file 1: Figure S2C). After *Cebpa* was overexpressed in hepatocytes, the APAP-ALI model was established. Increased serum ALT and AST levels suggested that *Cebpa* overexpression aggravated APAP-ALI (Fig. 4A), paralleling the images showing H&E staining (Fig. 4B) and the increase in the number of TUNEL-stained cells (Fig. 4C). Notably, *Cebpa* overexpression in hepatocytes induced the mRNA expression of *Il1b*, *Il6* and *Tnfa* in the liver (Fig. 4D). Moreover, we also detected increased *Cxcl1*, *Cxcl13* and *Mcp1* and decreased *Arg1* and *Il10* expression levels (Additional file 1: Figure S3A, B). For a survival study, we increased the dose of APAP administered (500 mg/kg) to the mice and found that *Cebpa* overexpression in the liver significantly increased the mortality of the mice treated with high-dose APAP (Additional file 1: Figure S3C).

IHC assay of liver sections showed that the expression of BMP9 was upregulated after *Cebpa* was overexpressed (Fig. 4E). Moreover, the rate of SMAD1/5/9 phosphorylation was increased (Fig. 4F). These results indicated that *Cebpa* overexpression promoted BMP9 production and activated BMP signaling. Next, we sought to identify the proteins that potentially control APAP-ALI progression in the liver after *Cebpa* is overexpressed. By performing immunoblotting, we confirmed that *Cebpa* was successfully overexpressed and that the expression level of BMP9 and SMAD1/5/9 phosphorylation rates were increased (Fig. 4G). Furthermore, we detected the downregulation of ATG3 and ATG7 expression in the liver, which was accompanied by blocked autophagy (Fig. 4H). In addition, we also detected increased *Bmp9* expression after successful *Cebpa* overexpression, followed by decreased *Atg3* and *Atg7* mRNA levels (Additional file 1: Figure S3D).

IF staining for F4/80 and BMP9 in liver sections revealed that *Cebpa* overexpression increased the expression of BMP9 localized to F4/80-positive cells in the liver (Fig. 4I). Similar to our observations of aged livers, increased expression of BMP9 in F4/80-negative cells was observed in livers after APAP treatment, suggesting the diversity and complexity of BMP9 sources in the liver (Fig. 4I).

### BMP9 inhibits ATG3 and ATG7 expression to block autophagy in MΦs

**Fig. 5:**
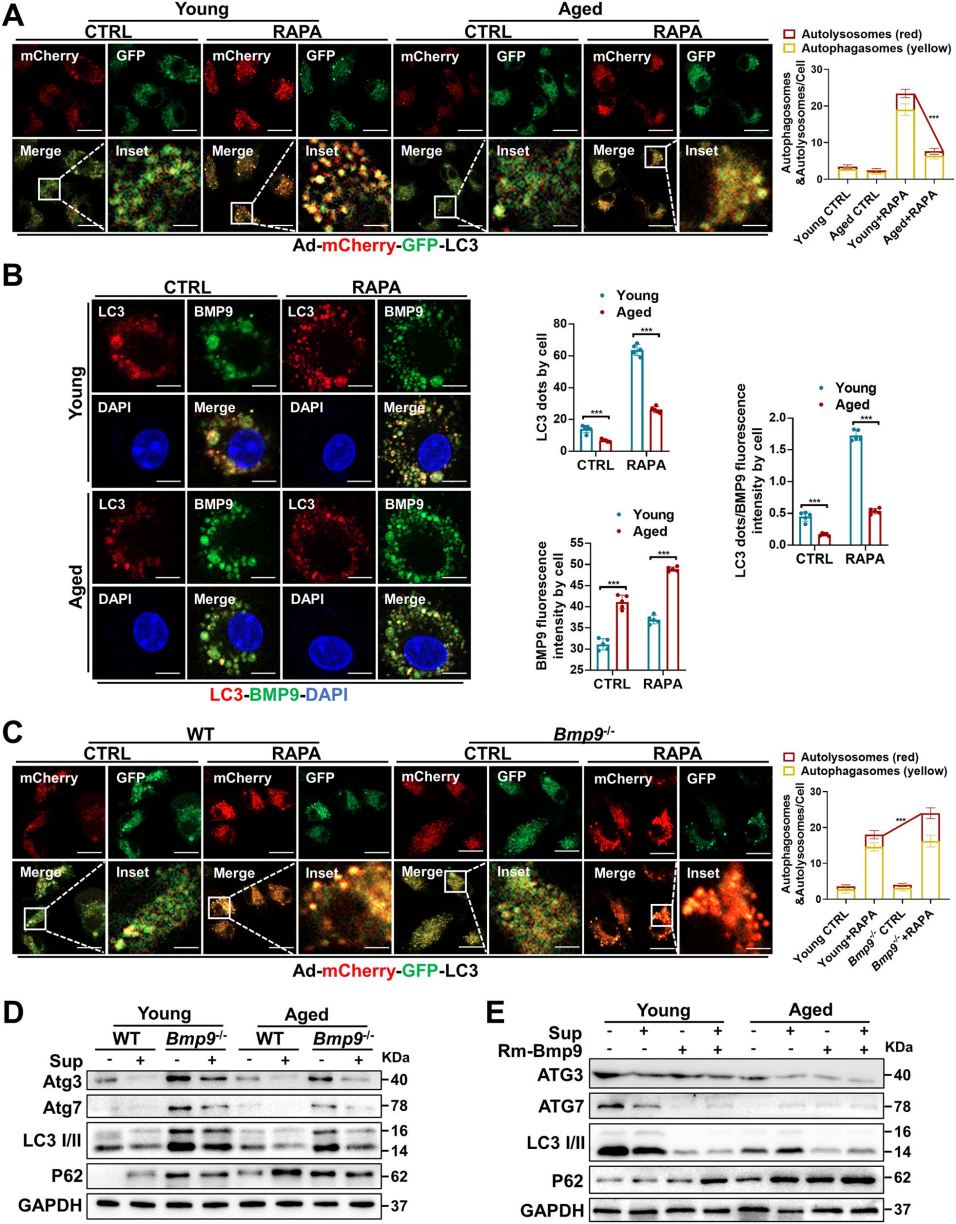
BMP9 inhibits ATG3 and ATG7 and thus blocks autophagy in MΦs. **A** BMDMs derived from the bone marrow of young and aged mice were infected with Ad-mCherry-GFP-LC3 (MOI = 20) on the fifth day of culture when the cells were not fully mature and then treated with rapamycin on the seventh day of culture. Representative confocal laser microscopy images of each group. Quantitative data are shown in the right panel. **B** BMDMs from young and aged mice were treated with rapamycin on day 7. Representative images showing IF staining for LC3-I/II (red) and BMP9 incorporation (green) with DAPI counterstaining (blue) in each group. Quantitative data are shown in the right panel. **C** BMDMs derived from the bone marrow of WT and *Bmp9*^*−/−*^ mice were infected with Ad-mCherry-GFP-LC3 (MOI = 20) on the fifth day of culture when the cells were not fully mature and then treated with rapamycin on the seventh day of culture. Representative confocal laser microscopy images of each group. Quantitative data are shown in the right panel. **D** Protein expression levels of ATG3, ATG7, LC3 I/II and p62 in BMDMs from WT and *Bmp9*.^*−/−*^ mice with or without APAP-treated AML-12 cell supernatant (Sup). **E** Protein expression levels of ATG3, ATG7, LC3 I/II and p62 were in BMDMs with or without Rm-BMP9 and/or Sup treatment. *p < 0.05, **p < 0.01, and ***p < 0.001

Our results thus far confirmed that BMP9 is abundantly expressed in hepatic MΦs and directly aggravates APAP-induced injury by inhibiting hepatocyte autophagy. Rapamycin (RAPA) has been extensively reported to activate autophagy and reduce senescence [19, 20]. Therefore, we investigated the roles of aging and BMP9 on MΦs treated with RAPA. The LC3 I-to-LC3 II conversion rate was reduced, and BMP9 was highly expressed in primary hepatic MΦs from aged mice treated with RAPA and/or bafilomycin (Ba) (Additional file 1: Figure S4). Next, after infecting Ad-mCherry-GFP-LC3 adenovirus and expressing mCherry-CFP-tagged LC3 in BMDMs, we confirmed that autophagy in aged mouse-derived MΦs was hindered compared with that in the young group (Fig. 5A). Dual IF staining for LC3 and BMP9 (two colors) was then performed with BMDMs derived from aged mice, and we found that the BMP9 level increased while LC3 level decreased in cells from the aged mice, as indicated through the assessment of high-resolution images (Fig. 5B). Furthermore, the number of mCherry puncta was increased in cells treated with RAPA after *Bmp9* was deleted (Fig. 5C).

Autophagy was inhibited in the short term after exposure to Sup (described above), which may be an important driver of MΦs proinflammatory action during the acute phase of liver injury (Fig. 5D, E). After BMP9 was depleted, ATG3 and ATG7 expression in MΦs was upregulated, the LC3 I-to-LC3 II conversion rate was increased, and the p62 level was decreased, indicating that autophagy had been activated (Fig. 5D). Additionally, Rm-BMP9 addition resulted in downregulated ATG3 and ATG7 expression, a decreased LC3 I-to-LC3 II conversion rate, and increased p62 accumulation in the MΦs (Fig. 5E).

In previous studies, a decline in autophagy promoted M1-type MΦ polarization during ALI, inducing proinflammatory and injury-promoting effects [21]. Importantly, BMP9 can directly promote APAP-induced hepatocyte death; therefore, the aging-associated excess accumulation of BMP9 may be an essential driver of aggravated liver injury.

### BMP9 regulates the senescence and immunophenotype acquisition of MΦs

After etoposide (1 μg/ml) treatment for 2 days, the mRNA levels of *p21* and *p53* in iBMDMs were increased, and the expression of the SASP components *Il1b*, *Il6* and *Tnfa* was upregulated (Additional file 1: Figure S5A). Similar to the BMP9 promotion of etoposide-induced hepatocyte senescence, BMP9 promoted etoposide-induced senescence phenotype acquisition by iBMDMs (Additional file 1: Figure S5B). In hepatic MΦs isolated from *Bmp9*^*−/−*^ mice, *Il1b*, *Il6*, *Tnfa* and *iNos* expression levels were reduced after etoposide treatment alone and/or with APAP-treated AML-12 cell supernatant treatment (Additional file 1: Figure S5C).

**Fig. 6:**
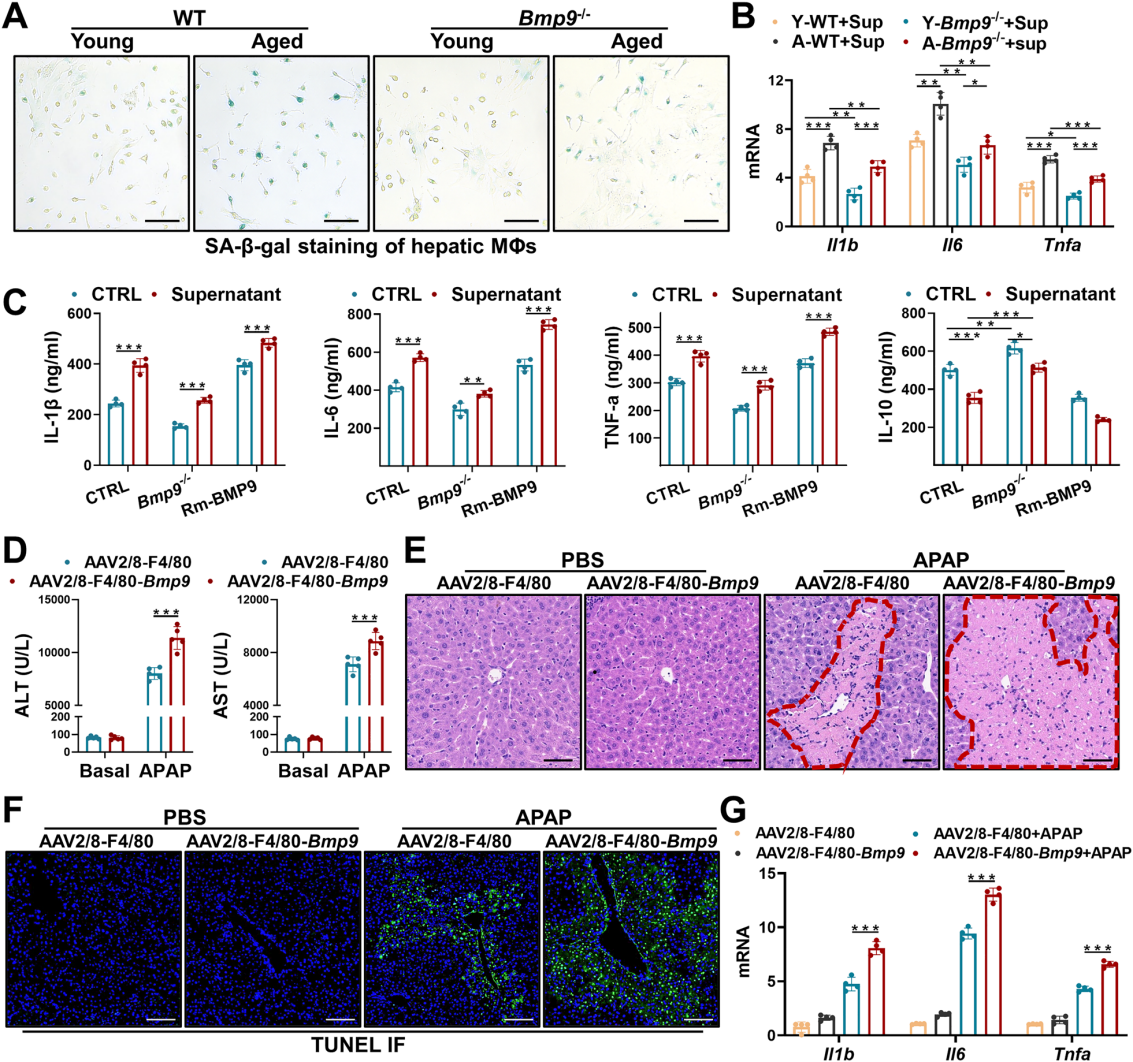
BMP9 regulates the senescence and immunophenotype acquisition of MΦs. **A** SA-β-gal staining of BMDMs derived from young and aged WT mice and young and aged *Bmp9*.^*−/−*^ mice. **B** mRNA expression levels of *Il1b*, *Il6* and *Tnfa* in each group. **C** Cytokine levels (IL-1β, IL-6, TNF-α and IL-10) in the supernatants of cells in each group as measured by ELISAs. To further study the effect of macrophage-derived BMP9 on liver injury, an F4/80-specific AAV2/8-overexpressing plasmid vector (AAV2/8-F4/80-*Bmp9*) was constructed. APAP-ALI (300 mg/kg) was established for 2 weeks after AAV2/8-F4/80-*Bmp9* and AAV2/8-F4/80-CTRL injection. Serum and liver tissue were collected from each group of mice 24 h later. **D**, **E** Serum ALT and AST levels (**D**) and representative images showing H&E staining (**E**) of liver slices of each group. **F** Representative images showing TUNEL staining. **G** mRNA expression levels of *Il1b*, *Il6* and *Tnfa*. The average target gene/*Gapdh* ratios of different experimental groups relative to the control group. *p < 0.05, **p < 0.01, and ***p < 0.001

Considering these results, we isolated primary hepatic MΦs from young WT and *Bmp9*^*−/−*^ mice and aged WT and *Bmp9*^*−/−*^ mice. SA-β-gal staining revealed that *Bmp9* deletion reduced the number of MΦs that acquired the senescence phenotype (Fig. 6A). Next, after these young and aged cells were treated with Sup, the expression levels of *Il1b*, *Il6* and *Tnfa* were decreased in the *Bmp9*^*−/−*^ group (Fig. 6B). Moreover, cytokine (IL-1β, IL-6, TNF-α and IL-10) levels in the supernatants of the cells subjected to different treatments were measured by ELISAs. The results showed that *Bmp9* deletion reduced the secretion of IL-1β, IL-6 and TNF-α (proinflammatory cytokines) and increased the production of IL-10 (a cytokine that suppresses inflammation and promotes injury repair), while increases in the exogenous Rm-BMP9 level significantly increased the levels of secreted IL-1β, IL-6 and TNF-α and reduced the concentration of IL-10 (Fig. 6C). The expression and intracellular distribution of iNOS and CD206 were then determined by IF staining, and the results showed that aging promoted iNOS production and decreased CD206 levels, and excess BMP9 in cells further enhanced iNOS production and reduced CD206 levels, while inhibiting *Bmp9* expression led to the opposite outcomes (Additional file 1: Figure S6A).

To further study the effect of MΦ-derived BMP9 on APAP-ALI, an F4/80-specific *Bmp9*-overexpressing AAV2/8 virus was constructed (AAV2/8-F4/80-*Bmp9*) [22]. APAP-ALI was established 2 weeks via tail vein injection of AAV2/8-F4/80-*Bmp9*. IF staining of isolated hepatic MΦs showed that BMP9 expression in the AAV2/8-F4/80-*Bmp9* group was significantly upregulated, confirming the successful overexpression of MΦ-specific BMP9 (Additional file 1: Figure S6B). As shown by serum ALT/AST levels and H&E staining, APAP-ALI severity was significantly increased in the F4/80-specific *Bmp9-*overexpressing group (Fig. 6D, E). Additionally, the number of TUNEL-stained cells in the livers of the F4/80-specific *Bmp9*-overexpressing mice treated with APAP were significantly increased (Fig. 6F). Moreover, the mRNA expression of *Il1b*, *Il6* and *Tnfa* was upregulated in the livers of the F4/80-specific *Bmp9*-overexpressing mice (Fig. 6G).

These data suggest that BMP9 plays a key role in the senescence of MΦs and that MΦ-derived BMP9 clearly drives the progression of APAP-ALI.

## Discussion

C/EBPα is involved in the regulation of myeloid cell differentiation, proliferation, metabolism and immune function [23] and has been reported to be involved in regulating the fate of all myeloid cells [24]. C/EBPα is required for maintaining the integrity of hematopoietic stem cells and plays a central role in the early stage of myeloid cell differentiation and maturation [25–27]. Importantly, the reprogramming of mature myeloid cells derived from bone marrow hematopoietic stem cells plays an indispensable role in regulating multiple physiological functions. C/EBPα in combination with PU1 or C/EBPβ reverses the acquisition of a lymphoid phenotype by B- or T-cell progenitors in vitro*,* and these progenitors ultimately differentiate into monocytes/granulocytes [28]. Treatment with therapeutic saRNA overexpressing C/EBPα (MTL-C/EBPα) in hepatic cancer patients indicated that the number of monocytic myeloid-derived suppressor cells (M-MDSCs) and M2 tumor-associated MΦs (TAMs) were significantly reduced [29], suggesting that C/EBPα attenuates the negative immunoregulatory function of MΦs after MΦ reprogramming. This outcome favors MΦ polarization s into tumor-killing M1 cells, whereas normal liver repair capacity is impaired after liver injury.

Our results in this study enabled us to identify a previously unknown BMP-involved mechanism of liver inflammation and injury in which C/EBPα regulates BMP9 expression and BMP9 further drives liver inflammation and injury via its effects on both hepatocytes and MΦs. No previous study had explored the function and mechanism of BMP9 in senescence-associated APAP-ALI; therefore, the similarities and differences between hepatocytes and MΦs have been unclear to date. However, previous studies have shown that the constitutively low level of BMP9 expression helps stabilize hepatocyte function and that excess BMP9 promotes liver injury and fibrosis [15]. Despite these findings, the precise role and mechanism of BMP9 in aging and APAP-ALI remain unclear. BMP9 is mainly expressed and secreted by the liver, and it is then redistributed throughout the body, where it exerts effects [13, 14]. Some studies have suggested that BMP9 is expressed mainly in hepatic nonparenchymal cells, such as hepatic stellate cells and KCs [13, 30], and other studies have shown that BMP9 is also expressed in hepatocytes [31]. Our results showed that BMP9 was constitutively expressed to a certain extent in MΦs in the resting state and that its expression in other cells (non-MΦs), including hepatocytes, was evident after APAP-ALI had been induced.

After BMPs induce receptor hetero-oligomerization, type I receptors are activated via the phosphorylation of type II receptors. These activated receptor complexes phosphorylate SMAD proteins (R-SMADs: SMAD1, SMAD5 and SMAD8/9), forming complexes with SMAD4 [30]. An R-SMAD complex enters the nucleus and regulates target gene expression by binding to a regulatory element and recruiting transcriptional corepressors and/or activation complexes to these promoters. In this study, enhanced BMP9 activated downstream SMAD1/5/9 phosphorylation, further mediating the downregulation of ATG3 and ATG7 and leading to autophagy suppression. Moreover, the study provides direct evidence suggesting that excess BMP9 leads to autophagy inhibition and is associated with a tendency toward increased APAP-ALI severity in aged mice. The degree of liver injury and the rate of proinflammatory phenotype acquisition were exacerbated when additional Rm-BMP9 was injected into APAP-ALI model mice and in livers treated with *Cebpa*-overexpressing AAV2/8 or F4/80-specific *Bmp9*-overexpressing AAV2/8. These results show that redundant BMP9 action promotes proinflammatory responses and liver injury. Moreover, severe liver injury and inflammatory responses were alleviated in *Bmp9*-ko mice treated with APAP.

By transporting cargo to be degraded to lysosomes, autophagosomes effectively removes excess or dysfunctional organelles and unwanted proteins, establishing favorable conditions for cell survival and maintenance of function. Two ubiquitin-like coupling systems, ATG12-ATG5 and LC3-phosphatidyl ethanolamine (PE) conjugates, are closely related to autophagosome formation. ATG12 is activated by ATG7 (an E1-like enzyme) ubiquitination, then transferred to ATG10 (an E2-like enzyme), and ultimately is attached to ATG5 to form a ATG5-ATG12 conjugate [17, 32]. This conjugate recruits ATG16L1 to form a multimeric complex that can enhance the formation of the LC3-PE conjugate [17]. ATG4 cleaves LC3 to generate LC3-I, which binds to PE through a ubiquitin-like reaction involving ATG7 and ATG3 (with E2-like enzyme activity) to form lipidated LC3 (LC3-II), which binds to the autophagosome membrane [18].

Among the multiple molecular changes associated with aging, altered autophagy has become a hallmark of senescence in different species, and autophagy deficiency during aging is one of the most common drivers of tissue homeostasis dysregulation [33]. Similarly, the regulation of inflammation is inextricably linked to autophagy, as impaired autophagy in immune cells leads to uncontrolled inflammation and is recognized as a major driver of aging-related tissue damage [34]. In previous studies, autophagy has been identified as a key mechanism that coordinates the metabolic and differentiation states of innate immune cells. When autophagic flux is inhibited, MΦs acquire a proinflammatory phenotype and promote tissue damage [33], and autophagy interventions can often achieve better therapeutic efficacy in aging-related diseases [19, 20]. Therefore, improving the resistance of elderly patients to liver injury has important clinical application value for the diagnosis and treatment of aging-related liver diseases.

Exploring the mechanism of senescence-associated MΦ reprogramming may help to identify its roles in senescence-associated liver injury, and hence, the development of personalized treatment regimens for liver diseases may lead to positive results. For example, intracellular oxidants inhibit ATG3 and ATG7 by inducing their oxidation, inhibiting autophagy and thereby promoting aging progression [35]. In this study, we revealed that BMP9 expression was increased in hepatocytes and MΦs in the livers of aged mice, while both ATG3 and ATG7 expression was decreased, disrupting autophagy. In cultured hepatocytes and MΦs, excessive levels of BMP9 downregulated the expression of ATG3 and ATG7, inhibiting autophagic flux. Further studies have indicated that when *Cebpa* was overexpressed in the liver, ATG3 and ATG7 were downregulated and autophagic flux was reduced, aggravating liver inflammation and injury.

## Conclusions

**Fig. 7:**
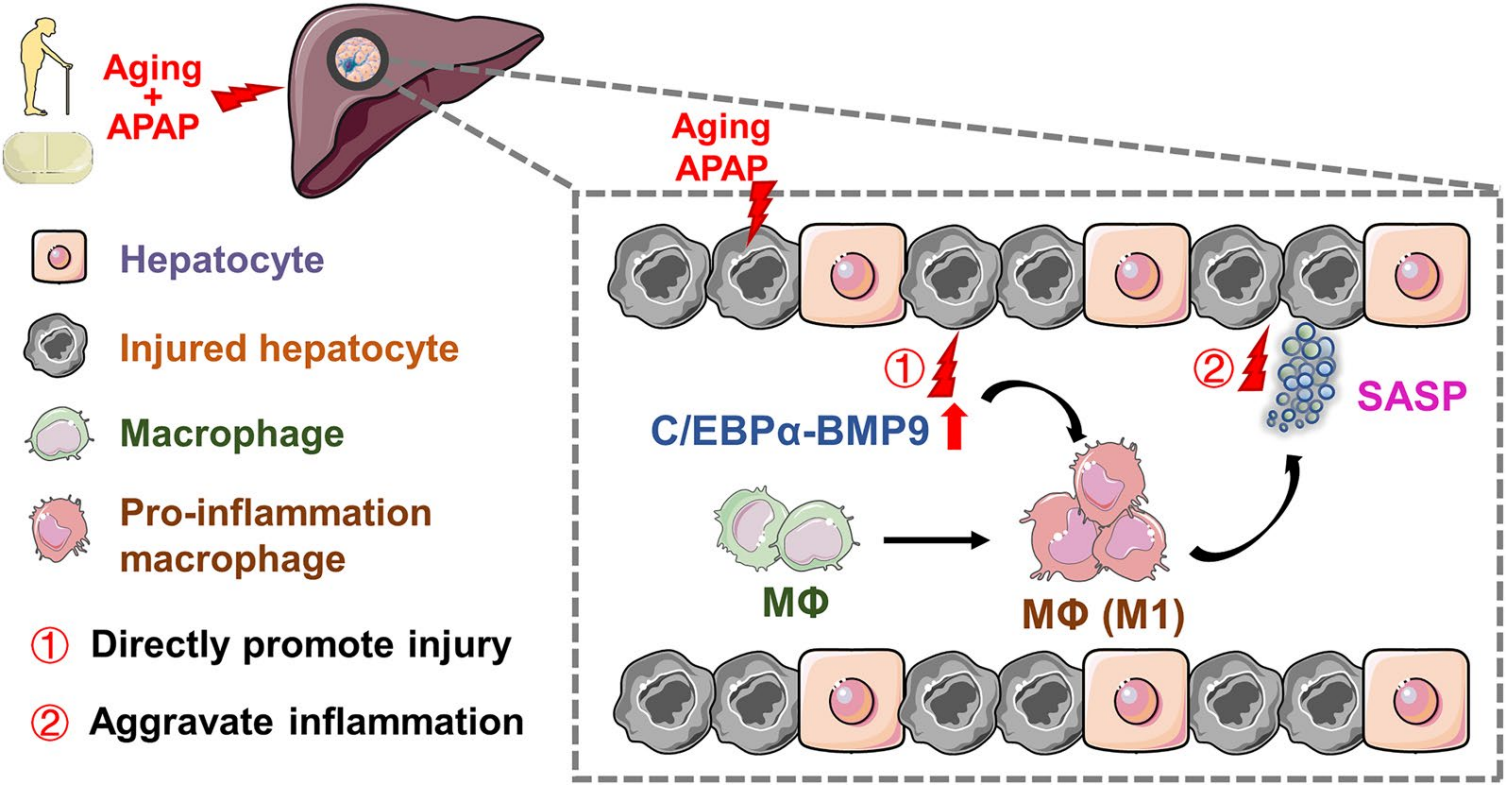
Model depicting the aggravation of ALI during aging through ectopic C/EBPα-BMP9 crosstalk

In conclusion, the excessive production of aging-associated C/EBPα upregulates BMP9 expression, which plays a central role in the aging-associated aggravation after APAP-ALI via its paracrine and autocrine activity. On the one hand, BMP9 directly accelerates APAP-induced hepatocyte death, and on the other hand, it promotes the proinflammatory phenotype acquisition of MΦs (Fig. 7). Mechanistically, BMP9 promotes the phosphorylation of the downstream transcription factors SMAD 1/5/9, which in turn leads to a decrease in ATG3 and ATG7 expression, which is associated with autophagy blockade. Our study revealed a crucial function for the C/EBPα-BMP9 axis in APAP-ALI and provided insight into acute liver injury. These results help in understanding the interrelatedness of different cells involved in acute liver injury and indicate new therapeutic directions. However, additional work is needed to identify how BMP9 interacts with regulatory signaling pathways in multiple cell types in the liver during aging and the common and unique effects of BMP9 on the senescence of other cells and tissues across the body.

## Materials and methods

### Animal experiments

Young (6–10 weeks) and old-aged (> 16 months) [36] male C57BL/6N mice were used in experiments. *Bmp9*^*−/−*^ mice were purchased from GemPharmatech Co., Ltd. The *Bmp9*^*−/−*^ mice with a C57BL/6 background were established by CRISPR‒Cas9, and the effects of treatments on KO mice were compared to those of WT littermates. All the uses of the mice were approved by the Laboratory Animal Ethics Committee of the University of Science and Technology of China. The mice were housed in an environmentally controlled, pathogen-free isolation facility under a 12-h light–dark cycle with food and water freely available. The mice were fasted overnight (i.e., for approximately 15 h) before the administration of 300 mg/kg APAP (30 μl/g body weight) or vehicle (warm PBS) via i.p. injection [37]. Liver injury was assessed via the determination of serum alanine aminotransferase (ALT) activity and hematoxylin and eosin (H&E) staining of liver Sects. [38].

### Cells

Mouse primary hepatocytes and MΦs were isolated from mice fed ad libitum via a combination of magnetic activated cell sorting and collagenase‐based density gradient centrifugation [39, 40]. The isolated hepatocytes were seeded on collagen-coated plates in Williams' E medium supplemented with 10% fetal bovine serum (FBS), 2 mm L-glutamine, 1% penicillin (100 U/ml)-streptomycin (100 μg/ml) (P/S), 100 nm dexamethasone, and 5% insulin transferrin selenium (ITS). A mouse hepatocyte cell line (AML12) was cultured in DMEM supplemented with 10% FBS, 5% ITS, 2 mm L-glutamine, 1% P/S, and 100 nm dexamethasone. Unless otherwise indicated, all cells were cultured at 37 °C in 95% ambient air and 5% CO_2_. Primary BMDMs were cultured for 6 days in DMEM supplemented with 10% FBS, m-CSF (20 ng/ml), and 1% P/S. iBMDMs were cultured in DMEM supplemented with 10% FBS and 1% P/S.

### Antibodies and reagents

The antibodies used in this study are listed in the Supplementary Information. Murine m-CSF (PeproTech, 315–02-100), Rm-BMP9 (R&D Systems, 5566-BP), RAPA (Sigma–Aldrich, V900930), and etoposide (Sigma–Aldrich, E1383) were obtained from commercially available sources.

Recombinant AAV2/8 was generated using pAAV-CR and pHelper in HEK293 cells as described previously [41]. AAV2/8 plasmid vectors overexpressing *Cebpa* were produced and purchased from Hanbio Tech (Shanghai, China). An empty vector was constructed at the same time. AAV2/8 plasmid vectors overexpressing *Bmp9* under the control of the MФ-specific AAV2/8-F4/80-*Bmp9* promoter were produced and purchased from Hanbio Tech (Shanghai, China). An empty vector under the control of the MФ-specific AAV2/8-F4/80 promoter was constructed at the same time. AAV2/8-F4/80-*Bmp9* titers of 1.5 × 10^13^ vector genomes (vg)/mL and AAV2/8-F4/80 titers of 1.5 × 10^13^ vg/mL were used in the study.

### Immunohistochemistry, immunofluorescence, and immunoblotting

The immunohistochemistry, immunofluorescence and immunoblotting experiments are described in detail in the Supplementary Information.

### Plasmids, siRNAs, and Rt-qPCR

Cell lines (the AML12 and iBMDM cell lines) were transfected with overexpression (OE) plasmids (OE-*Bmp9* and OE*-Cebpa*) (Shanghai GeneChem Co., Ltd.), or small interfering RNAs (siRNAs) (*Bmp9* siRNA and si*Cebpa* siRNA) (Tsingke Biotechnology Co., Ltd.) using Lipofectamine 3000 (Invitrogen, L3000). Total RNA was extracted using TRIzol (Invitrogen). We synthesized cDNA from 1 μg of total RNA following the manufacturer’s protocol (Takara, RR036A). Then, RT–PCR was performed with a premixed kit (Takara, RR820A) according to the manufacturer’s instructions. The sequences of the primer pairs are listed in Additional file 1: Table S1.

### Ad-mCherry-GFP-LC3

Ad-mCherry-GFP-LC3 (Beyotime Biotechnology, C3011), which is an adenovirus expressing a mCherry-GFP-LC3 fusion protein, was used for autophagy detection after infection of cells or tissues as described previously [42]. BMDMs were infected with Ad-mCherry-GFP-LC3 (MOI = 20) according to the manufacturer's instructions on the fifth day of culture, when the cells were not fully mature, and then treated with LPS or Torin 1 (1 µM) on the seventh day of culture.

### Statistical analysis

Statistical analysis was performed with Prism 8.2 (GraphPad Software). Specifically, one-way analysis of variance (ANOVA) was performed for comparisons among groups. The differences in survival based on survival curves were determined via log-rank test. Differences for which P < 0.05 were considered to be statistically significant.

## Supplementary Information

**Additional file 1: Table S1. **Primer sequences used for real-time RT-PCR. **Figure S1. **Supplementary figure related to Figure 2. (A-B) Rm-BMP9 was injected into the tail vein, the APAP-ALI model was established 1 h later, and 24 h later, serum and liver tissue were collected from each group of mice. Representative images showing TUNEL staining (A) and the mRNA expression levels of related cytokines (*Il1b*, *Il6* and *Tnfa*) (B) in each group. (C-D) An APAP-ALI model was established with young and aged WT mice and with young and aged *Bmp9*^*-/-*^ mice. Representative images showing TUNEL staining (C) and the mRNA expression levels of related cytokines (*Il1b*, *Il6* and *Tnfa*) (D) in each group. (E) IF staining for P-SMAD1/5/9 in young and aged WT mice and aged *Bmp9*^*-/-*^ mice. The average target gene/*Gapdh* ratios of different experimental groups relative to the control group. GAPDH was used as the loading control for immunoblotting. *p < 0.05, **p < 0.01, and ***p < 0.001. **Figure S2. **BMP9 expression is regulated by C/EBPα *in vivo*. (A-C) *Cebpa*-overexpressing AAV2/8 was injected into the mouse tail vein 2 weeks after the models were established. (A) IF and IHC staining for C/EBPα in liver slices. (B) mRNA expression levels of *Cebpa *and *Bmp9*. (C) Protein expression levels of C/EBPα and BMP9. The average target gene/*Gapdh* ratios of different experimental groups relative to the control group. GAPDH was used as the loading control for immunoblotting. *p < 0.05, **p < 0.01, and ***p < 0.001. **Figure S3. **Supplementary figures related to Figure 4. (A-B) Additional mRNA expression level measurements (*Cxcl1*, *Cxcl13*, *Mcp1*, *Arg1* and *Il10*). (C) Survival curves of mice in the Cebpa-overexpressing and control groups when the dose of APAP treatment was increased to 500 mg/kg. (D) mRNA expression level measurements (*Cebpa*,* Bmp9*,* Atg3* and *Atg7*). The average target gene/*Gapdh* ratios of different experimental groups relative to the control group. *p < 0.05, **p < 0.01, and ***p < 0.001. **Figure S4.** (A) LC3 I/II and BMP9 levels in primary hepatic MΦs from young and aged mice treated with APAP-treated AML-12 cell supernatant (Sup) and/or RAPA and/or BA. GAPDH was used as the loading control for immunoblotting. **Figure S5. **BMP9 increases etoposide-induced macrophage senescence and proinflammatory cytokine expression. (A) mRNA expression levels of *p21*, *p53,* *Il1b*, *Il6* and *Tnfa*. (B) SA-β-gal staining for iBMDMs after control treatment, etoposide treatment for 1 day, etoposide treatment for 2 days, or etoposide plus Rm-BMP9 treatment for 2 days. (C) mRNA expression levels of *iNos*, *Il1b*, *Il6* and *Tnfa *in hepatic MΦs isolated from *Bmp9*^*-/-*^ and WT mice. The average target gene/*Gapdh* ratios of different experimental groups relative to the control group. *p < 0.05, **p < 0.01, and ***p < 0.001. **Figure S6. **(A) Representative images showing IF staining for iNOS (red) and CD206 incorporation (green) with DAPI counterstaining (blue) in each iBMDM group. (B) Representative images showing BMP9 IF staining of primary MΦs isolated from mouse livers treated after AAV-F4/80 or AAV-F4/80-*Bmp9* injection.

## Data Availability

All data and material will be made available on request.
