# Supplementary material for: Aging aggravates acetaminophen-induced acute liver injury and inflammation through inordinate C/EBPα-BMP9 crosstalk
Source: Cell Biosci. 2023 Mar 21;13:61. doi: 10.1186/s13578-023-01014-6 (PMC10029235; doi:10.1186/s13578-023-01014-6)
Supplement: Supplementary file 1 — Additional file 1: Table S1. Primer sequences used for real-time RT-PCR. Figure S1. Supplementary figure related to Figure 2. (A-B) Rm-BMP9 was injected into the tail vein, the APAP-ALI model was established 1 h later, and 24 h later, serum and liver tissue were collected from each group of mice. Representative images showing TUNEL staining (A) and the mRNA expression levels of related cytokines (Il1b, Il6 and Tnfa) (B) in each group. (C-D) An APAP-ALI model was established with young and aged WT mice and with young and aged Bmp9-/- mice. Representative images showing TUNEL staining (C) and the mRNA expression levels of related cytokines (Il1b, Il6 and Tnfa) (D) in each group. (E) IF staining for P-SMAD1/5/9 in young and aged WT mice and aged Bmp9-/- mice. The average target gene/Gapdh ratios of different experimental groups relative to the control group. GAPDH was used as the loading control for immunoblotting. *p < 0.05, **p < 0.01, and ***p < 0.001. Figure S2. BMP9 expression is regulated by C/EBPα in vivo. (A-C) Cebpa-overexpressing AAV2/8 was injected into the mouse tail vein 2 weeks after the models were established. (A) IF and IHC staining for C/EBPα in liver slices. (B) mRNA expression levels of Cebpa and Bmp9. (C) Protein expression levels of C/EBPα and BMP9. The average target gene/Gapdh ratios of different experimental groups relative to the control group. GAPDH was used as the loading control for immunoblotting. *p < 0.05, **p < 0.01, and ***p < 0.001. Figure S3. Supplementary figures related to Figure 4. (A-B) Additional mRNA expression level measurements (Cxcl1, Cxcl13, Mcp1, Arg1 and Il10). (C) Survival curves of mice in the Cebpa-overexpressing and control groups when the dose of APAP treatment was increased to 500 mg/kg. (D) mRNA expression level measurements (Cebpa, Bmp9, Atg3 and Atg7). The average target gene/Gapdh ratios of different experimental groups relative to the control group. *p < 0.05, **p < 0.01, and ***p < 0.001. Figure S4. (A) LC3 I/ [file 13578_2023_1014_MOESM1_ESM.docx]

**Additional file 1: Information**

**Aging aggravates acetaminophen-induced acute liver injury and inflammation through inordinate C/EBPα-BMP9 crosstalk**

Rui Liu^1,3^, Wentao Xu^3^, He Zhu^1^, Zijian Dong^4^, Huke Dong^4^, Shi Yin^1,2^

^1^Department of Geriatrics, Affiliated Provincial Hospital of Anhui Medical University, Anhui Medical University, Hefei 230001, China

^2^Department of Geriatrics, the First Affiliated Hospital of USTC, Division of Life Sciences and Medicine, University of Science and Technology of China, Hefei, Anhui 230001, China

^3^Department of Oncology, the First Affiliated Hospital of Anhui Medical University, Hefei 230022, China

^4^Clinical Medical College of Anhui Medical University, Hefei 230036, China

Correspondence: Shi Yin, M.D., Ph.D., Department of Geriatrics, Affiliated Provincial Hospital of Anhui Medical University, Anhui Medical University, Hefei, Anhui 230001, PR China. E-mail: drshiyin@ustc.edu.cn (S. Yin)

Rui Liu, Wentao Xu and He Zhu contributed equally in this study

**Supplementary Materials and Methods**

**Immunohistochemistry**

Liver sections of a 4 µm thickness that were fixed in 4% paraformaldehyde buffered with PBS were used for staining. After deparaffinization, the sections were washed in PBS 3 times. EDTA buffer and Dako blocking reagent were used for heat-induced antigen retrieval and blocking of endogenous peroxidase activity. Primary antibody incubation was performed at 4 °C overnight with shaking, followed by streptavidin-conjugated horseradish peroxidase antibody incubation at room temperature for 2 hours. DAB staining was performed, and the sections were washed with distilled water, followed by hematoxylin counterstaining.

**Immunofluorescence**

Cells were fixed on slides with 4% paraformaldehyde for 10 min. After washing with PBS three times, the cells were permeabilized with 0.5% Triton X-100 in PBS for 10 min. After rinsing the cells with PBS, with cells were blocked with PBS containing 0.025% Triton X-100 and 1% BSA for 1 hour at room temperature. The slides were then incubated with the primary antibodies in 0.025% Triton X-100 and 1% BSA in PBS at 4 °C overnight. Then, the slides were washed with PBS with 0.025% Triton X-100 three times and incubated with fluorochrome-conjugated secondary antibody and DAPI for 1 hour. The images were obtained on a Zeiss Axio Observer 3 imaging system or Zeiss LSM 800 confocal laser scanning microscope.

**Immunoblotting**

Total protein extraction was performed with RIPA buffer supplemented with complete protease inhibitor cocktail. The lysates were clarified by centrifugation at 12000 rpm for 15 min at 4 °C, and the concentration was measured by a Nanodrop 2000 (Thermo Fisher). The lysate was denatured at 100 °C for 10 min and then mixed with a loading buffer to 1X. Protein extracts (5–10 μl) were separated on 10% SDS-polyacrylamide gels and transferred onto PVDF membranes. Then, blocking with 5% nonfat milk in TBST (20 mM Tris base, 137 mM NaCl, 0.1% Tween 20, pH 7.4) was performed for 1 h at room temperature. The membrane was incubated overnight at 4 °C in a 5% BSA-TBST solution containing the following primary antibodies: LC3-I/II (Cell Signaling Technology, 12741), ATG3 (Cell Signaling Technology, 3415), ATG7 (Cell Signaling Technology, 8558), P62 (Cell Signaling Technology, 16177), iNOS (Cell Signaling Technology, D6B6S), CD206 (Santa Cruz, sc-70586), BMP9 (Santa Cruz, sc-514211), C/EBPα (Cell Signaling Technology, 8178), SMAD1 (Proteintech, 10429-1-AP), PSMAD1/5/9 (Cell Signaling Technology, 13820), and GAPDH (Cell Signaling Technology, 5174).

**Table S1.** Primer sequences used for real-time RT-PCR

| *Gene* (Mouse) | *Forward/Reverse* | *Primer (5’ to 3’)* |
| --- | --- | --- |
| *Gapdh* | Forward | ATGTTCCAGTATGACTCCACTCACG |
|  | Reverse | CAGCTTCTCCACAGCCACAA |
| *Il1b* | Forward  Reverse | TTGACGGACCCCAAAAGATG TGGATGCTCTCATCAGGACAG |
| *Il6* | Forward  Reverse | CCAGAAACCGCTATGAAGTTCC CGGACTTGTGAAGTAGGGAAGG |
| *Tnfα* | Forward  Reverse | GGTGCCTATGTCTCAGCCTCTT GCCATAGAACTGATGAGAGGGAG |
| *Il10* | Forward  Reverse | GCTCTTACTGACTGGCATGAG CGCAGCTCTAGGAGCATGTG |
| *Mcp1* | Forward  Reverse | TCATGCTTCTGGGCCTGCTG TCTCATTTGGTTCCGATCCAGGTT |
| *iNos* | Forward | GTTCTCAGCCCAACAATACAAGA |
|  | Reverse | GTGGACGGGTCGATGTCAC |
| *Arg1* | Forward | CCACAGTCTGGCAGTTGGAAG |
|  | Reverse | GGTTGTCAGGGGAGTGTTGATG |
| *Bmp9* | Forward | CAGAACTGGGAACAAGCATCC |
|  | Reverse | GCCGCTGAGGTTTAGGCTG |
| *Cebpa* | Forward | GCAAAGCCAAGAAGTCGGTG |
|  | Reverse | CACCTTCTGTTGCGTCTCCA |
| *p21* | Forward | TCCACAGCGATATCCAGACA |
|  | Reverse | GGACATCACCAGGATTGGAC |
| *p53* | Forward | AGCTTTGAGGTTCGTGTTTGTG |
|  | Reverse | TGGGCAGCGCTCTCTTTG |
| *Cxcl1* | Forward | TCCAGAGCTTGAAGGTGTTGCC |
|  | Reverse | AACCAAGGGAGCTTCAGGGTCA |
| *Cxcl13* | Forward | CATAGATCGGATTCAAGTTACGCC |
|  | Reverse | GTAACCATTTGGCACGAGGATTC |


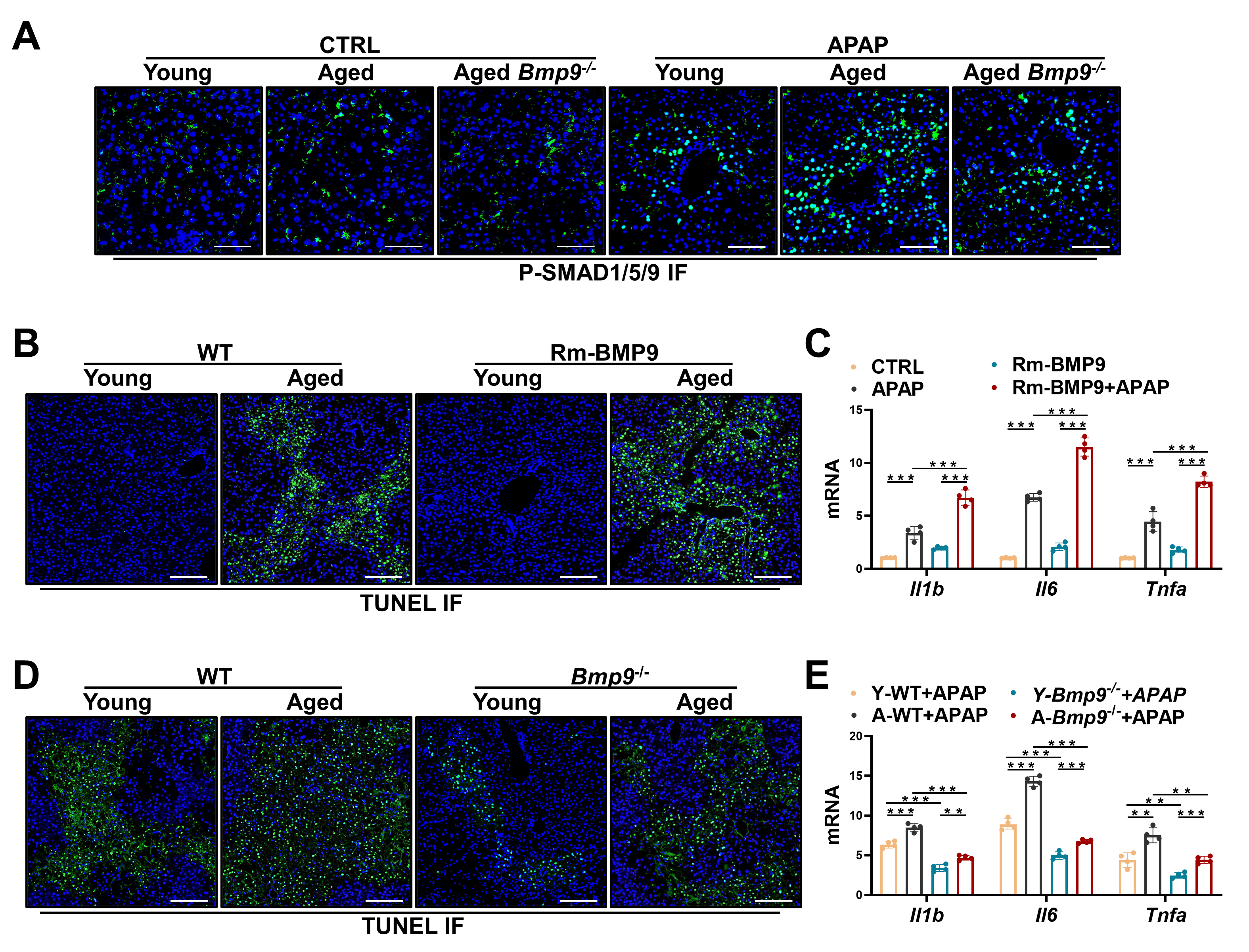


**Figure S1.** Supplementary figure related to Figure 2. (A-B) Rm-BMP9 was injected into the tail vein, the APAP-ALI model was established 1 h later, and 24 h later, serum and liver tissue were collected from each group of mice. Representative images showing TUNEL staining (A) and the mRNA expression levels of related cytokines (*Il1b*, *Il6* and *Tnfa*) (B) in each group. (C-D) An APAP-ALI model was established with young and aged WT mice and with young and aged *Bmp9^-/-^* mice. Representative images showing TUNEL staining (C) and the mRNA expression levels of related cytokines (*Il1b*, *Il6* and *Tnfa*) (D) in each group. (E) IF staining for P-SMAD1/5/9 in young and aged WT mice and aged *Bmp9^-/-^* mice. The average target gene/*Gapdh* ratios of different experimental groups relative to the control group. GAPDH was used as the loading control for immunoblotting. *p < 0.05, **p < 0.01, and ***p < 0.001.


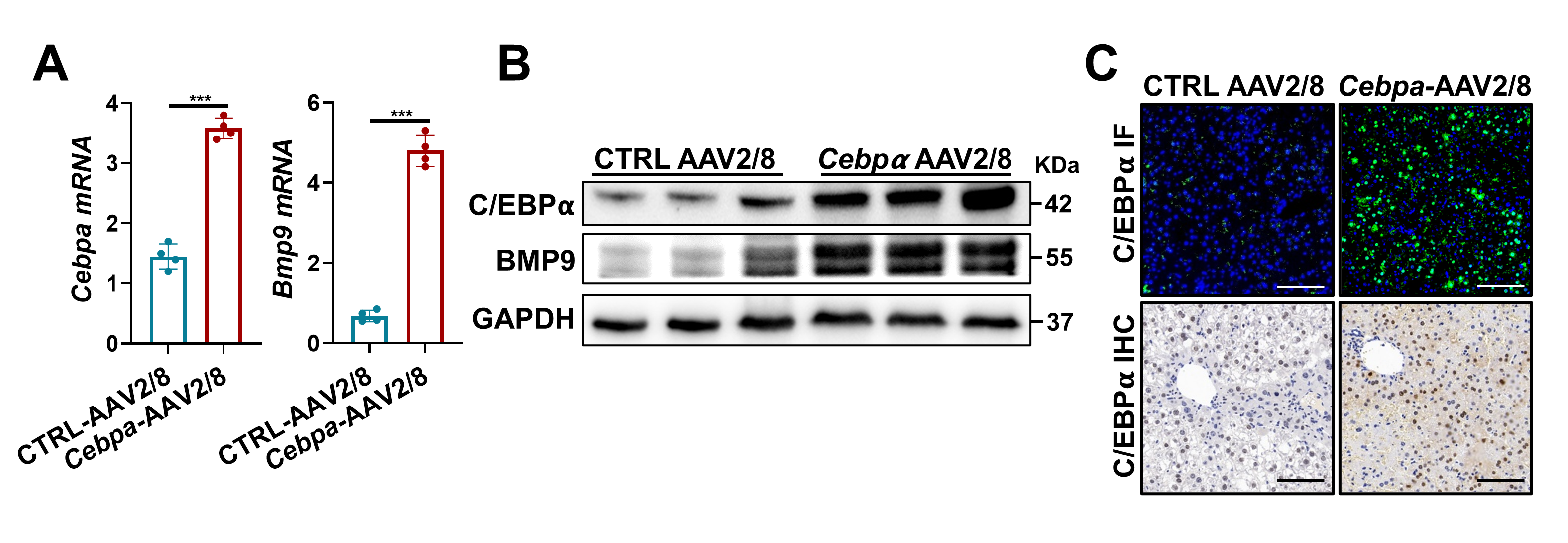


**Figure S2.** BMP9 expression is regulated by C/EBPα *in vivo*. (A-C) *Cebpa*-overexpressing AAV2/8 was injected into the mouse tail vein 2 weeks after the models were established. (A) IF and IHC staining for C/EBPα in liver slices. (B) mRNA expression levels of *Cebpa* and *Bmp9*. (C) Protein expression levels of C/EBPα and BMP9. The average target gene/*Gapdh* ratios of different experimental groups relative to the control group. GAPDH was used as the loading control for immunoblotting. *p < 0.05, **p < 0.01, and ***p < 0.001.


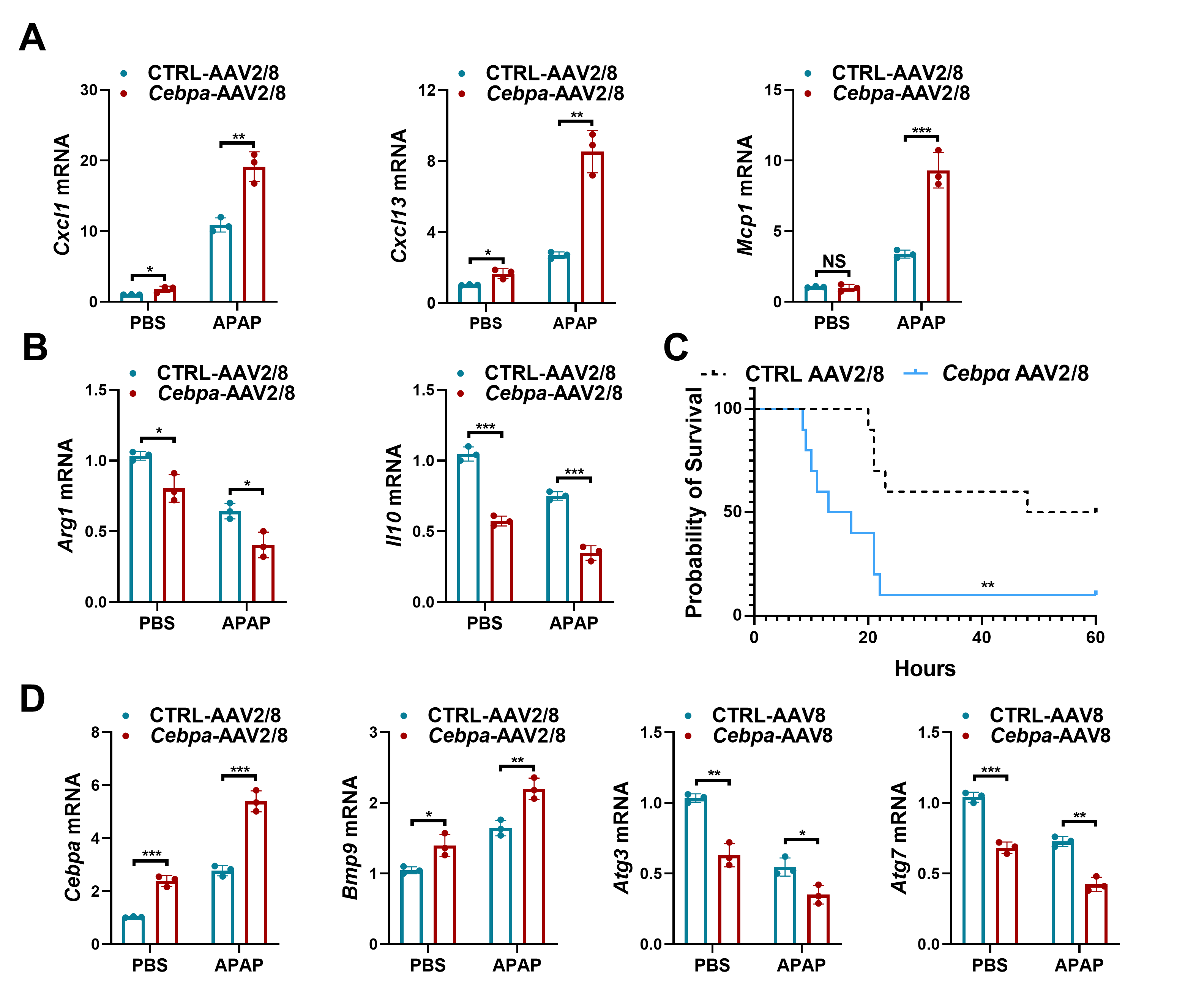


**Figure S3.** Supplementary figures related to Figure 4. (A-B) Additional mRNA expression level measurements (*Cxcl1*, *Cxcl13*, *Mcp1*, *Arg1* and *Il10*). (C) Survival curves of mice in the Cebpa-overexpressing and control groups when the dose of APAP treatment was increased to 500 mg/kg. (D) mRNA expression level measurements (*Cebpa*, *Bmp9*, *Atg3* and *Atg7*). The average target gene/*Gapdh* ratios of different experimental groups relative to the control group. *p < 0.05, **p < 0.01, and ***p < 0.001.


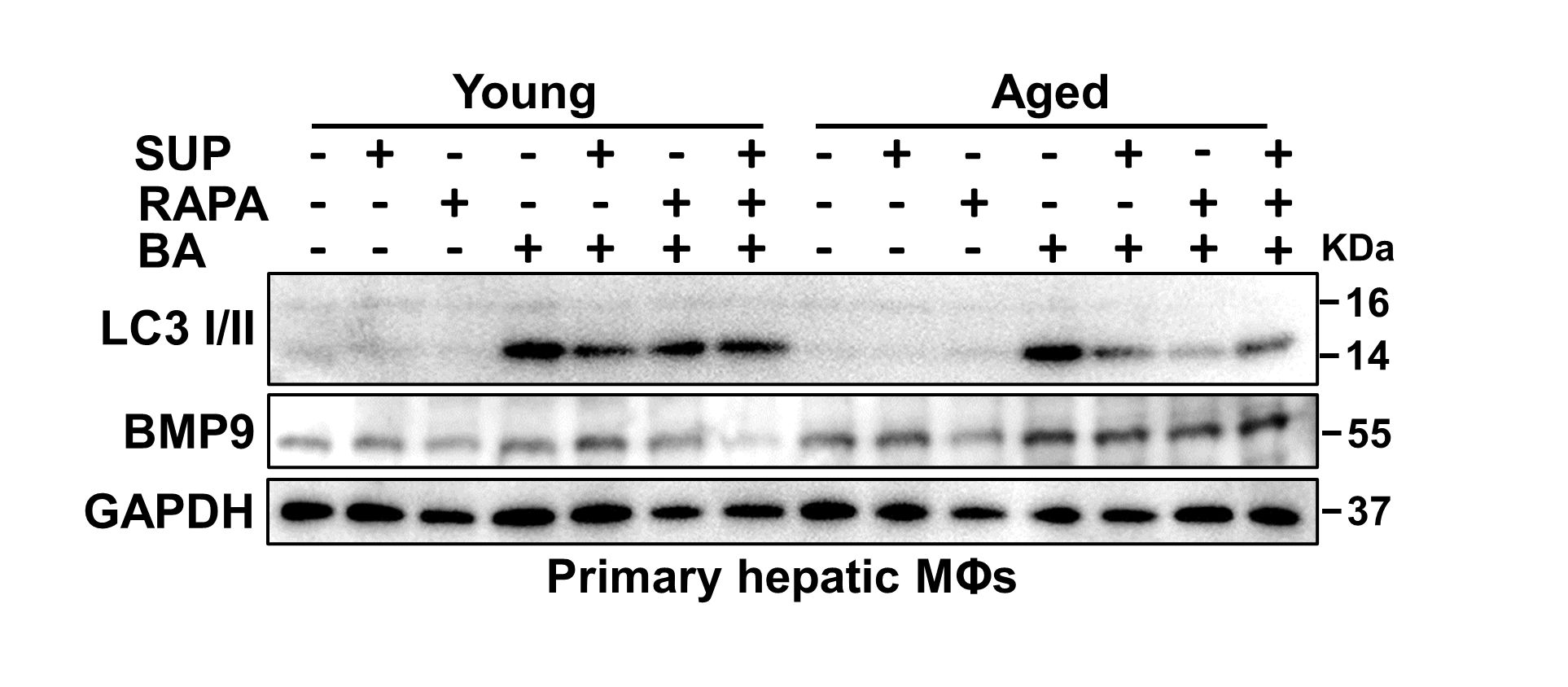


**Figure S4.** (A) LC3 I/II and BMP9 levels in primary hepatic MΦs from young and aged mice treated with APAP-treated AML-12 cell supernatant (Sup) and/or RAPA and/or BA. GAPDH was used as the loading control for immunoblotting.


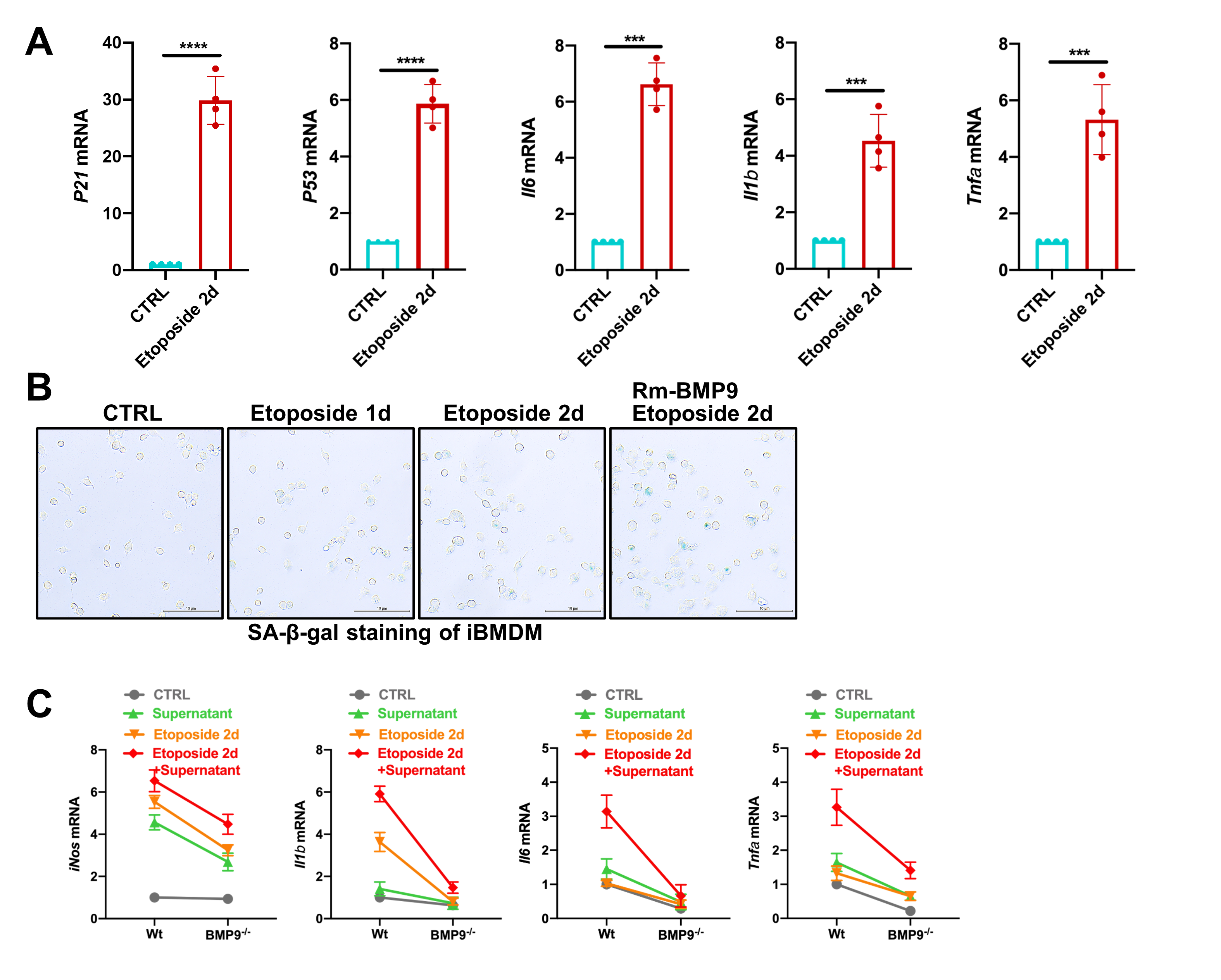


**Figure S5.** BMP9 increases etoposide-induced macrophage senescence and proinflammatory cytokine expression. (A) mRNA expression levels of *p21*, *p53,* *Il1b*, *Il6* and *Tnfa*. (B) SA-β-gal staining for iBMDMs after control treatment, etoposide treatment for 1 day, etoposide treatment for 2 days, or etoposide plus Rm-BMP9 treatment for 2 days. (C) mRNA expression levels of *iNos*, *Il1b*, *Il6* and *Tnfa* in hepatic MΦs isolated from *Bmp9^-/-^* and WT mice. The average target gene/*Gapdh* ratios of different experimental groups relative to the control group. *p < 0.05, **p < 0.01, and ***p < 0.001.


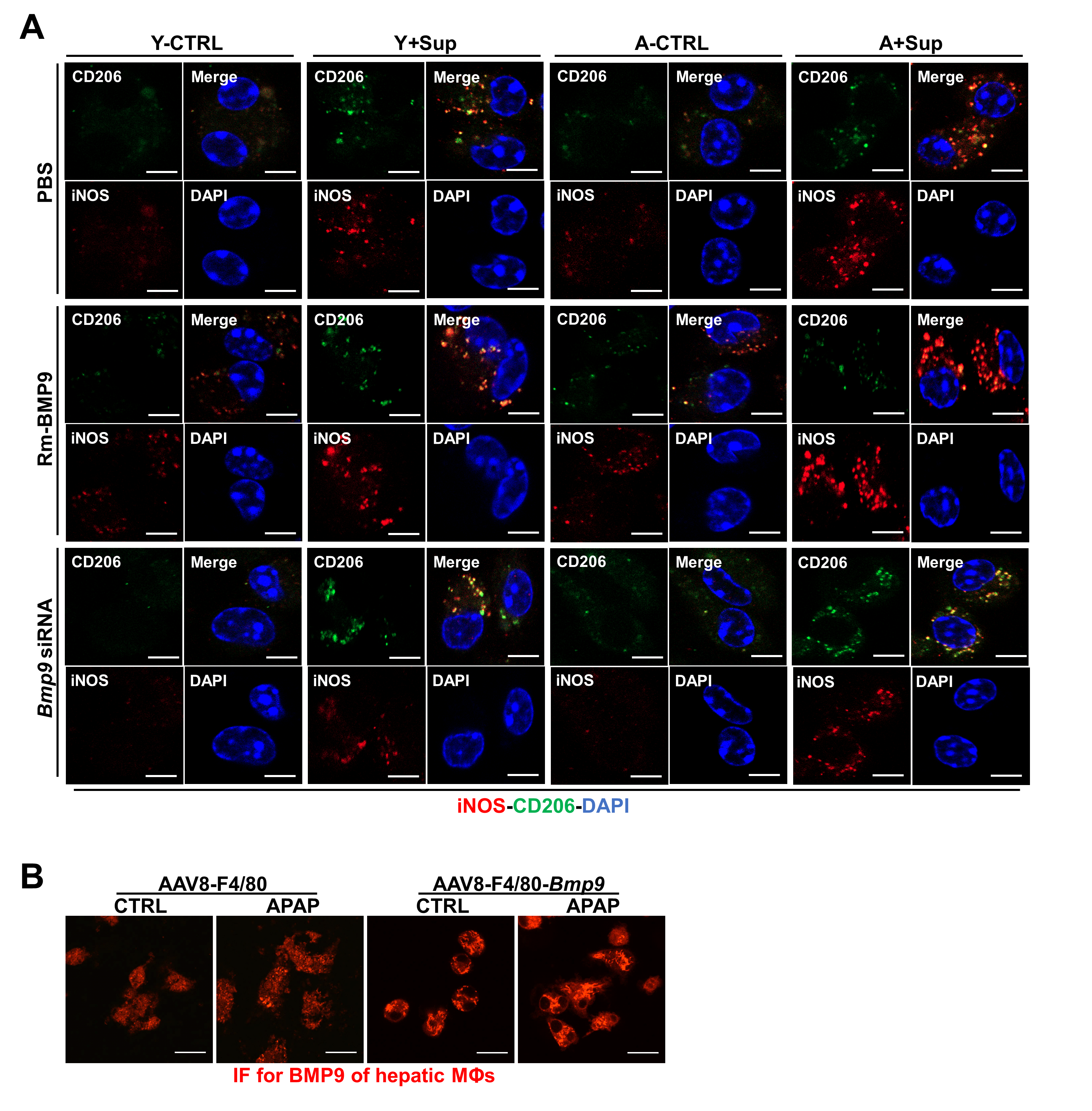


**Figure S6.** (A) Representative images showing IF staining for iNOS (red) and CD206 incorporation (green) with DAPI counterstaining (blue) in each iBMDM group. (B) Representative images showing BMP9 IF staining of primary MΦs isolated from mouse livers treated after AAV-F4/80 or AAV-F4/80-*Bmp9* injection.
